# Who and Where? The Spatial Context of Racial and Ethnic Disparities in Economic Security among Older Adults

**DOI:** 10.1093/geroni/igab046.2374

**Published:** 2021-12-17

**Authors:** Yang Li, Jan Mutchler, Nidya Velasco Roldan

**Affiliations:** 1 Institute of Social Sciences; 2 University of Massachusetts Boston, Boston, Massachusetts, United States; 3 University of Massachusetts, Boston, Massachusetts, United States

## Abstract

Many older adults experience challenging financial circumstances and do not have sufficient income to afford a basic budget in their home communities. Far higher proportions of racial and ethnic minority older adults live on incomes that fall short of what is needed to make ends meet relative to their White counterparts. We describe racial/ethnic disparities in late-life economic insecurity, which occurs when an older person lacks sufficient financial resources to cover necessary expenses in their home community. Although nationwide half of older singles are economically insecure, Massachusetts (62%), New York (65%), Vermont (57%), and Mississippi (57%) have the highest shares of older adults who experience economic insecurity. Compared to Whites, minority older adults have higher rates of economic insecurity in nearly every state, but racial/ethnic disparities are higher in some locations (Rhode Island, Massachusetts, Mississippi, Louisiana) and lower in others (Oregon, Arizona, Nevada, West Virginia). Disparities in economic insecurity reflect the precarious financial situations experienced by many older adults, rooted not only in risks and disadvantages accumulated over time, but also in the variable and uncertain social and economic contexts that accompany the aging experience. By situating older adults in their places of residence, we observe that the cost of remaining in community intersect with life-course experiences associated with social identities to produce disparities in economic security at older ages. The geographic variation in cost of living calls for context-specific assessment of economic security to evaluate the adequacy of economic resources and the associated risk of hardship.

